# COGTIPS: a double-blind randomized active controlled trial protocol to study the effect of home-based, online cognitive training on cognition and brain networks in Parkinson’s disease

**DOI:** 10.1186/s12883-019-1403-6

**Published:** 2019-07-31

**Authors:** Tim D. van Balkom, Henk W. Berendse, Ysbrand D. van der Werf, Jos W. R. Twisk, Iris Zijlstra, Rob H. Hagen, Tanja Berk, Chris Vriend, Odile A. van den Heuvel

**Affiliations:** 1 Amsterdam UMC, Vrije Universiteit Amsterdam, Psychiatry, Amsterdam Neuroscience, De Boelelaan 1117, Amsterdam, Netherlands; 2 Amsterdam UMC, Vrije Universiteit Amsterdam, Anatomy and Neurosciences, Amsterdam Neuroscience, De Boelelaan 1117, Amsterdam, Netherlands; 3 Amsterdam UMC, Vrije Universiteit Amsterdam, Neurology, Amsterdam Neuroscience, De Boelelaan 1117, Amsterdam, Netherlands; 4 Amsterdam UMC, Vrije Universiteit Amsterdam, Epidemiology and Biostatistics, Amsterdam Public Health, De Boelelaan 1117, Amsterdam, Netherlands; 5 Dutch Parkinson’s Disease Association, PO Box 46, Bunnik, 3980 CA the Netherlands

**Keywords:** Parkinson’s disease, Cognitive training, Cognitive rehabilitation, Cognitive impairment, Neuropsychological assessment, Neuroimaging, MRI, Network, RCT

## Abstract

**Background:**

Cognitive dysfunction is highly prevalent in Parkinson’s disease (PD) and a large proportion of patients eventually develops PD-related dementia. Currently, no effective treatment is available. Cognitive training is effective in relieving cognitive dysfunctions in several –neurodegenerative– diseases, and earlier small-scale trials have shown positive results for PD. In this randomized controlled trial, we assess the efficacy of online home-based cognitive training, its long-term effects, as well as the underlying neural correlates in a large group of PD patients.

**Methods:**

In this double-blind randomized controlled trial we will include 140 non-demented patients with idiopathic PD that experience significant subjective cognitive complaints. Participants will be randomized into a cognitive training group and an active control group. In both groups, participants will individually perform an online home-based intervention for eight weeks, three times a week during 45 min. The cognitive training consists of thirteen games that focus on executive functions, attention and processing speed with an adaptive difficulty. The active control comprises three games that keep participants cognitively engaged without a training component. Participants will be subjected to extensive neuropsychological assessments at baseline and after the intervention, and at six months, one year and two years of follow-up. A subset of participants (40 in each treatment condition) will undergo structural and functional magnetic resonance imaging. The primary outcome of this study is the performance on the Tower of London task. Secondary outcomes are objective and subjective cognitive functioning, conversion to PD-related mild cognitive impairment or dementia, functional and structural connectivity and network topological indices measured with magnetic resonance imaging. None of the outcome measures are part of the cognitive training program. Data will be analyzed using multivariate mixed-model analyses and odds ratios.

**Discussion:**

This study is a large-scale cognitive training study in PD patients that evaluates the efficacy in relieving cognitive dysfunction, and the underlying mechanisms. The strengths of this study are the large sample size, the long follow-up period and the use of neuroimaging in a large subsample. The study is expected to have a low attrition and a high compliance rate given the home-based and easily-accessible intervention in both conditions.

**Trial registration:**

ClinicalTrials.gov ID NCT02920632. Registered September 30, 2016.

**Electronic supplementary material:**

The online version of this article (10.1186/s12883-019-1403-6) contains supplementary material, which is available to authorized users.

## Background

### Background and rationale

Cognitive impairments are among the plethora of non-motor symptoms associated with Parkinson’s disease (PD) [1, 2]. Approximately 25% of PD patients suffer from significant cognitive impairments already at the time of diagnosis [3, 4], and up to 80% eventually develop PD dementia (PD-D) [5, 6]. Moreover, compared with people without PD, patients with PD have up to 5.9 times the risk to develop dementia [7]. Cognitive impairments have a negative impact on performing the activities of daily living [8, 9] and are an important modulator in the development of neuropsychiatric symptoms, including psychosis [10, 11]. Degeneration of dopaminergic and non-dopaminergic systems is one of the alleged causes of cognitive impairments [12, 13] and have therefore been targets for pharmacological treatments. Although these drugs have modest temporary effects on cognitive symptoms by improving the attentional capacity, they have no proven efficacy in preventing further cognitive decline in PD [14, 15]. Hence, non-pharmacological treatment options must be considered as an alternative treatment for alleviating cognitive dysfunction in PD.

#### Cognitive training in PD: the gap in knowledge

Cognitive training (CT) was developed after the first brain tumor resections and traumatic brain injury treatment during the World Wars [16], but is currently applied in numerous neurological and psychiatric diseases. Meta-analyses have confirmed its efficacy in relieving cognitive dysfunction in Alzheimer’s disease [17], mild cognitive impairment (MCI) [18], schizophrenia [19], and traumatic brain injury [20, 21]. Furthermore, a recent meta-analysis in PD yielded positive results of CT mainly in relieving ‘frontal’ cognitive dysfunction (i.e. executive dysfunction, and working memory and psychomotor speed impairment) [22]. This meta-analysis, however, included only seven randomized controlled trials (RCTs), with a maximum sample size of 73 PD patients [23]. Consequently, the authors called for larger trials in PD populations – a conclusion that had earlier been stated in a systematic review [24] – although the results cautiously implied cognitive training to be efficacious.

#### The potential of cognitive training to preserve and protect

**Fig. 1:**
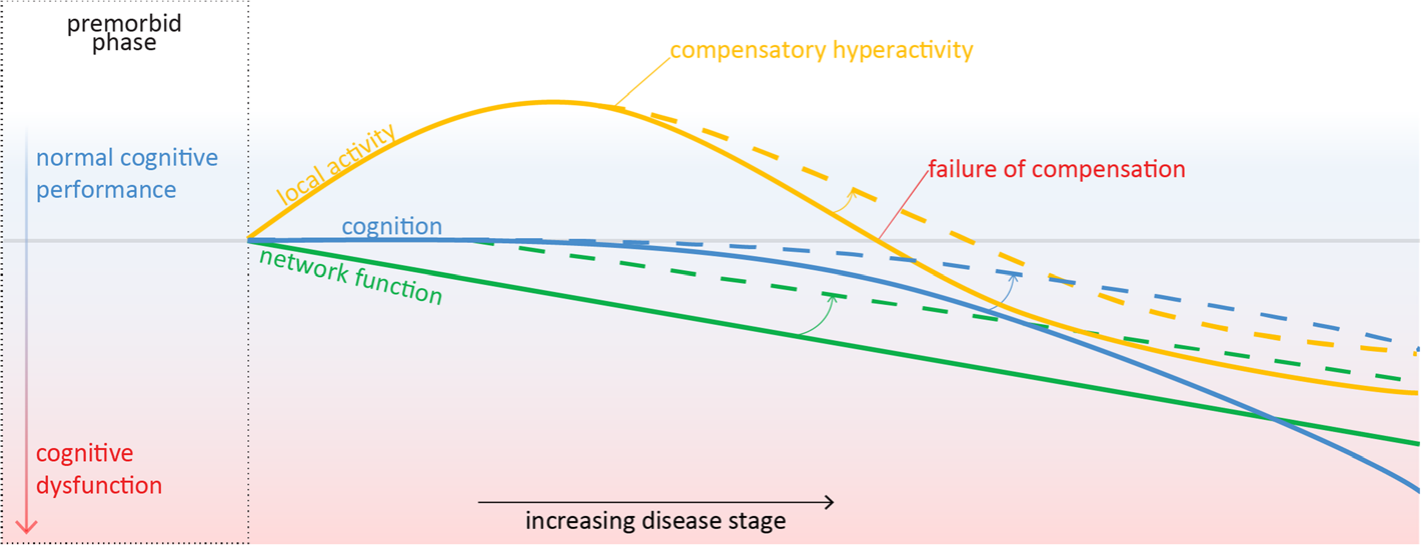
Working model of local compensatory brain activity (in yellow) that preserves intact cognitive functioning (in blue) but fails at later disease stage, while global brain network integrity gradually degenerates (in green). Dashed lines illustrate the hypothesized effects that CT may have on local and global brain infrastructure and on cognitive function. Adapted from [37]

Two study protocols have recently been published, describing a cognitive training intervention in PD [25, 26]. Both interventions are specifically aimed at patients who have already developed PD-related MCI [26] or PD-D [25], respectively. However, neural changes have been demonstrated early on in cognitively preserved PD [27–30]: at this stage compensatory local hyperactivity seems to counteract the progressive buildup of PD pathology that threatens global brain network function [31, 32]. At a later disease stage, this compensatory mechanism gradually fails and ultimately leads to brain-wide network failure and cognitive dysfunction [33–35]. An early-stage intervention to boost the compensatory phase during this *window of opportunity* is imperative to try and preserve cognitive functions and protect patients from cognitive decline (for a working model, adapted from [36], see Fig. 1).

Cognitive training may induce reorganization of structural and functional networks in the brain: it has been proposed that CT leaves a ‘footprint’ on the brain, that prepares the brain for better and faster processing [37]. Multiple studies have provided evidence that CT can induce reorganization of the brain network infrastructure. For example, patients with amnestic MCI showed post-CT normalization of within- and between-network connectivity [38, 39] that correlated with improved performance on memory tasks [39]. In addition, CT can alter resting-state networks in multiple sclerosis [40–42], normalize task-related activity in patients with schizophrenia [43, 44], and enhance functional connectivity [37, 45, 46] and cerebral blood flow [37] in healthy elderly. To date, only a few reports have focused on the underlying neural alterations after CT in PD [47–49] in small and mainly exploratory studies (*N* = 10–30). Results were mixed, showing increased functional connectivity [48], increased local activation [47, 48], but also decreased local activation [49] in comparison with controls.

In this study we aim to assess the efficacy of CT in a large sample of PD patients using a longitudinal design. Moreover, we aim to establish working mechanisms of CT by visualizing the within- and between-network changes that occur during training and to use the pre-treatment network topology, combined with the demographic and clinical characteristics, to predict who will profit most from CT.

## Methods and design

### Study objectives

In this study protocol we present COGTIPS – the “COGnitive Training In Parkinson Study”. The main research questions of this project are 1) What is the short-term and long-term effect of CT on objective and subjective cognitive functioning in PD? and 2) What are the neural mechanisms underlying the effect of CT in PD?

The study objectives of the COGTIPS study involve assessing an easily-accessible, home-based cognitive function training in individuals with mild subjective cognitive complaints in PD. Our *primary objective* is to assess the efficacy of an online CT program (compared to an active control condition) on executive functions. Our *secondary objectives* are to evaluate CT compared with an active control condition (AC) on 1) the efficacy on relieving subjective cognitive complaints; 2) the durability of the effect after 6 months, 1 year and 2 years; 3) the rate of conversion to PD-MCI and PD-D after 1 year and 2 years; 4) the effect on brain network efficiency and connectivity. Furthermore, we aim to identify baseline brain network characteristics that predict treatment outcome.

Based on previous literature on CT in PD and other neurodegenerative diseases, we hypothesize that compared with an active control condition 1) CT alleviates cognitive –mainly executive– dysfunction in PD patients, 2) CT relieves subjective cognitive complaints in daily-life, 3) the CT effect endures for up to 2 years after finishing the intervention, and reduces the risk of conversion to PD-MCI and PD-D, and 4) CT improves brain network efficiency and connectivity.

### Study design and setting

COGTIPS is a monocenter phase-III randomized controlled trial that will enroll one-hundred-and-forty (140) PD patients. To assess the superiority of the online CT compared with an AC, participants are randomly appointed to either of the conditions in a 1:1 fashion (70 versus 70). Eighty participants (i.e. 40 in each condition) will undergo pre- and post-training neuroimaging to assess CT-specific effects on functional and structural connectivity. This study was approved by the VU University Medical Center Medical Ethical Committee and this protocol is reported in accordance with SPIRIT guidelines (see SPIRIT checklist in Additional file 2) [50].

The COGTIPS study will be performed at the Amsterdam University Medical Centers (Amsterdam UMC), location VUmc, an academic hospital with expertise in movement disorders located in Amsterdam, the Netherlands. We will enroll Dutch-speaking PD patients that have shown their interest in participation through 1) the outpatient clinic for movement disorders of the Amsterdam UMC, or community or academic hospitals in the area, 2) the PD patient association (*“Parkinson Vereniging”*), 3) advertisements in media like the Parkinson Magazine and national newspapers, 4) advertisements on participant recruiting websites such as ‘ParkinsonNext’ and ‘Hersenonderzoek.nl’, and 4) a database of PD patients that have previously shown interest in online cognitive training.

### Eligibility criteria

Participants will be included on the basis of the presence of subjective cognitive complaints. We will focus on mild-to-moderate disease stage PD patients with mild cognitive complaints, to ensure that these patients are still within the ‘window of opportunity’. An overview of the inclusion and exclusion criteria is depicted in Table 1.

**Table 1:** Overview of inclusion and exclusion criteria

Inclusion criterion	Measured with	Defined by
Significant subjective cognitive complaints	Parkinson’s Disease Cognitive Functional Rating Scale	Score > 3
Mild to moderate disease stage	Hoehn & Yahr disease stage	Score < 4
Access to computer or tablet with access to Internet. Capability to use keyboard and computer mouse	Phone interview	–
Signed informed consent	–	–
**General exclusion criterion**	**Measured with**	**Defined by**
Indication for dementia syndrome	Self-administered Gerocognitive Examination	Score < 14
	Montreal Cognitive Assessment	Score < 22
Current drug- or alcohol abuse	CAGE AID-interview	Score > 1
Inability to undergo extensive neuropsychological assessments or eight weeks of home-based cognitive intervention	–	–
Moderate to severe depressive symptoms	Beck depression inventory	Score > 18
Presence of one or more impulse control disorders	ICD criteria interview	Positive screening
Psychotic symptoms. Benign hallucinations with insight are not an exclusion criterion	Schedule for Assessment of Positive Symptoms – PD	Positive screening
Traumatic brain injury	Phone interview	Cerebral contusion with 1) loss of consciousness for > 15 min and 2) posttraumatic amnesia > 1 h
**Exclusion criterion for participation in magnetic resonance imaging**	**Measured with**	**Defined by**
A space occupying lesion	Assessment by radiologist	–
Significant vascular abnormalities	Assessment by radiologist	Fazekas > 1
Severe claustrophobia	MRI safety screening questionnaire	Positive screening
Presence of metal in the body (e.g. pacemaker, neurostimulator)		
Pregnancy		
Difficulty with, or shortness of breath during 60 min of lying still		

### Participant timeline

**Fig. 2:**
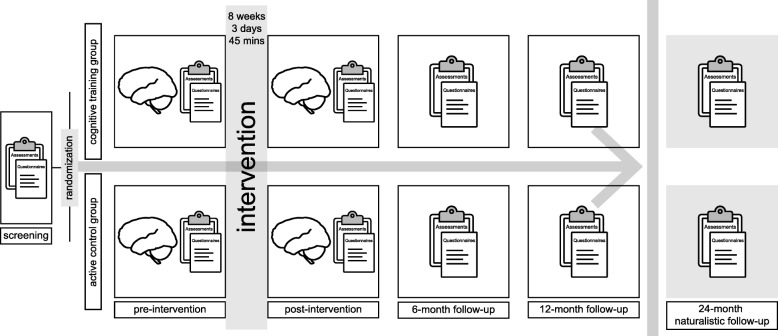
Global overview of the COGTIPS time schedule

Figure 2 shows a global overview of the time schedule. A detailed description of the participant visits and assessments is shown in Table 2.

**Table 2:** Tabular overview of the study time schedule including assessments and visits

Time-point	T-2	T-1	T0	T1	T2	T3	T4
Pre-screening							
Informed consent for pre-screening	X						
SAGE	X						
PD-CFRS	X			X	X	X	X
MRI safety screening	X						
Alcohol abuse screening (CAGE-AID)	X						
Eligibility screening							
Montreal Cognitive Assessment		X		X	X	X	X
ICD diagnostic criteria		X		X			
SAPS-PD^†^		X					
Beck depression inventory		X		X	X	X	X
Hoehn & Yahr stage		X				X	X
Enrolment and allocation			X				
Intervention							
Cognitive training			←→				
Active control condition			←→				
Assessments							
*Neuropsychological assessment*							
1	Tower of London		X	X	X	X	X
	Montreal Cognitive Assessment^a^			X	X	X	X
	Pentagon copy		X	X	X	X	X
1/2	Stroop Color Word Test		X	X	X	X	X
1	COWAT (‘letter fluency’) ^a^		X	X	X	X	X
2	WAIS-III digit span		X	X	X	X	X
3	Rey Auditory Verbal Learning Test^b^		X	X	X	X	X
3	Location Learning Test^c^		X	X	X	X	X
4	Boston naming test		X	X	X	X	X
4	Category fluency		X	X	X	X	X
5	Rey Complex Figure Test		X	X	X	X	X
5	Visual Form Discrimination Test		X	X	X	X	X
*Questionnaires and interviews*							
	CFQ		X	X	X	X	X
	Apathy scale		X	X	X	X	X
	Parkinson anxiety scale		X	X	X	X	X
	QUIP-RS		X	X	X	X	X
	NZPAQ-SF		X	X	X	X	X
	Credibility/expectancy questionnaire		X				
*Motor symptom assessments*							
	UPDRS-III - motor score		X			X	X
*Medication use*							
	Levodopa equivalent daily dosage		X	X	X	X	X
Neuroimaging*							
	MP-RAGE		X	X			
	3D PSIR		X	X			
	fMRI - resting state		X	X			
	DTI		X	X			

*Cognitive domains*: ^1^Executive functioning, ^2^Attention and working memory, ^3^Memory, ^4^Language, ^5^Visuospatial. *Abbreviations*: *CFQ* Cognitive Failures Questionnaire, *COWAT* Controlled Oral Word Association Test, *DTI* diffusion tensor imaging, *MP RAGE* magnetization-prepared 180 degrees radio-frequency pulses and rapid gradient-echo; (f) *MRI* (functional) magnetic resonance imaging, *NZPAQ-SF* New Zealand Physical Activity Questionnaire – Short Form, *PD-CFRS* Parkinson’s Disease – Cognitive Functional Rating Scale, *PSIR* phase-sensitive inversion recovery, *QPE* Questionnaire for Psychotic Experiences, *QUIP-RS* Questionnaire for Impulsive-Compulsive Disorders in Parkinson’s Disease – Rating Scale, *SAPS-PD* Scale for Assessment of Positive Symptoms for Parkinson’s disease, *UPDRS* Unified Parkinson’s Disease Rating Scale, *WAIS* Wechsler Adult Intelligence Scale

An overview of cognitive assessments and questionnaires, including references is provided in additional file 1

*in a subsample of *N* = 80

Parallel forms of the same test are used at consecutive visits if available: ^a^Three parallel forms; ^b^Two parallel forms; ^c^One parallel form

#### Pre-screening, screening and baseline assessment

PD patients that have shown interest in participating in COGTIPS will first undergo pre-screening for which they are required to sign informed consent and send this back by mail or E-mail. Pre-screening consists of a self-administered cognitive screening and questionnaires that are filled out at home (i.e. Self-administered Gerocognitive Examination [51]), and a phone interview. Patients are asked whether they are interested in participating in the subgroup that will undergo neuroimaging and if so, are screened for contraindications. After positive pre-screening, eligible patients are invited for an intake measurement.

At intake, patients will sign informed consent for participation in COGTIPS. They first undergo face-to-face screening of cognitive dysfunction by the Montreal Cognitive Assessment [52, 53], motor impairment by the Unified Parkinson’s Disease – Rating Scale part III [54], psychotic symptoms by the Schedule for Assessment of Positive Symptoms – PD [55], depressive symptoms by the Beck Depression Inventory [56]) and impulse control disorders (ICDs) by an ICD criteria interview. Eligible patients will undergo the baseline assessment (‘T0’) which comprises an extensive neuropsychological assessment, structured interviews and questionnaires. A sub-population will undergo magnetic resonance imaging. Neuroimaging data will be acquired at the Amsterdam UMC, location VUmc, on a Discovery* MR750 3.0 T MRI scanner (General Electric, Milwaukee) with a 32-channel head coil. We will obtain structural imaging (i.e. T1 and diffusion tensor imaging) and functional resting-state imaging. See Additional file 1 for the scan parameters. All assessments are performed by study members that are blinded for the treatment condition. The screening and baseline assessment will be performed during a single visit to the Amsterdam UMC, location VUmc.

#### Condition allocation and instructions

Following a positive screening for eligibility, a non-blinded study member will allocate the participant to either the CT or AC condition. Participants will be consecutively assigned to either the CT or AC condition on the basis of a randomization sequence. The randomization sequence is generated in Microsoft Excel by using computer-generated random numbers. We will use stratified randomization in which two strata will be generated according to education level. Vocational education level (or lower) defined as an education level of 5 or lower according to a Dutch classification system [57], which is comparable to 11 or less years of education [58]. High education level is defined as level 6 or 7 according to the Verhage classification system, which is comparable to 12 or more years of education.

A non-blinded study member will provide instructions to the participant concerning the log-in procedure for the training, the various training components, and the duration and frequency of training. After instructions, the participant will be asked to fill out a questionnaire concerning the patients’ expectations and credibility regarding the intervention [59]. Participants will additionally receive a hand-out with instructions to take home.

#### Eight-week intervention period

After the baseline assessment, participants may directly start with the 8-week intervention. A detailed description of the CT and AC interventions is provided below. Compliance will be monitored automatically and will be checked weekly. During the intervention, patients will receive biweekly questionnaires to ensure compliance and check for questions and problems performing the intervention. Non-blinded study members will follow-up on potential problems by phone.

#### Post-intervention assessments

After 24 intervention sessions, patients are invited for the post-intervention assessment. This assessment will be scheduled as close as possible to the last training session. Participants will first evaluate the intervention with a non-blinded study member. Directly afterwards, participants will undergo a post-intervention assessment (‘T1’). This assessment comprises a neuropsychological assessment and questionnaires (see Table 2). One team member (TB) will be de-blinded after the last T1 visit. All assessments after baseline will make use of parallel versions of neuropsychological tasks, if possible.

After 6 months (‘T2’), 1 year (‘T3’) and 2 years (‘T4’), participants will again undergo an extensive neuropsychological assessment and questionnaires. At T3 and T4, motor symptoms will also be assessed. From T3 onwards will be a naturalistic follow-up.

#### Blinding

Outcome assessors will be blinded for the full length of their role as assessor, while non-blinded team members will not assess participants at any point in this study. Blinded study members will not have access to the key of the randomization. Trial participants will be blinded for the full length of the study. Participants will be asked not to share any details of their intervention with the outcome assessor at any point in the study. When the participants’ condition is revealed to an outcome assessor, he or she will be replaced by another assessor for this participant.

#### Drop-outs

Participants that drop out of the study after being allocated to an intervention condition will not be replaced. We expect a low drop-out rate on the basis of our pilot study (one drop-out in 21 participants) and the low burden and short duration of both training conditions. In our sample size calculation, we conservatively account for 10% drop-out.

In case a participant withdraws from the study after 4 weeks of training (or more), we will aim to schedule an exit-measurement to measure the intervention effect.

#### Medication adjustments

Participants and their neurologist will be requested to retain a stable medication regime during the study period, specifically during the intervention. Patients and their neurologist will be requested to inform the study team if medication changes are clinically necessary.

### Interventions

The intervention in this study aims to train cognitive abilities, with a focus on executive functions, working memory, attention, and processing speed. A modified version of the BrainGymmer online CT platform (https://www.braingymmer.com/en/, a product by Dezzel Media B.V.) is used to provide the training at the patients’ home. We selected this method of cognitive training as it has been evaluated positively in our earlier pilot study in PD patients (see below), it is accessible for patients at home, and previous versions have been used in prior studies [60, 61]. A proof-of-concept in 20 PD patients showed that the experimental condition was evaluated as feasible and enjoyable. Moreover, the CT compared with an active control showed a medium interaction effect size on an executive functioning composite (i.e. Stroop Color Word Test, Trail Making Test and Controlled Oral Word Association Test), with a significantly positive change of executive functioning in the CT group but not in the active controls. Specifically, a large positive interaction effect size of CT on the Stroop color word test was found compared with controls (see Additional file 1 for a visual representation).

#### Intervention characteristics

In both conditions, 24 training sessions are performed: three times a week for a length of 8 weeks. The training sessions last approximately 45 min, marginally dependent on the participants’ performance. Compliance and training performance data are automatically tracked when a participant performs a training session. Participants can independently schedule the three training sessions per week to ensure flexibility and a low training threshold. The training sessions can be paused at the participants’ discretion but they are advised to try and complete the entire training within 1 hour.

#### Cognitive training

In the experimental condition, 13 CT games are sequentially performed. The cognitive processes that the training games call upon are similar to processes that are tested during the neuropsychological assessments, but the games are substantially different from the neuropsychological tasks. The training games are equipped with a ‘dynamic difficulty adjustment’: the difficulty of training components is adaptive to the participants’ performance, and will increase or decrease depending on individual performance. This way, participants will be challenged to continuously perform at their maximal ability. Training games, their duration and the hypothesized cognitive loading are shown in Table 3.

**Table 3:** Description of training games in the CT condition with their duration and the cognitive loading

Description	Duration	Cognitive loading
Repeat a drum rhythm that increases in length	3 mistakes	Working memory, attention
Flanker task	80 s	Cognitive flexibility
Put a sequence in the correct prompted order	180 s	Visuospatial function, focused attention
An ‘N-back’ task using bottles of various shapes and colors	180 s	Working memory
Evaluate if a ‘totem pole’ comprising blocks of different forms and diameters matches a top view	2 mistakes	Visuospatial function, mental rotation
Follow one or more moving targets (i.e. a bunny with a carrot) between several distractors	4 mistakes	Focused and divided attention
Accept or decline stimuli based on switching rules with increasing speed	90 s	Cognitive flexibility, processing speed
Remember an increasing number of colored squares	120 s	Working memory, attention
Click an increasing number of stimuli (i.e. food on a barbeque) at the right time (i.e. when they are well-done)	180 s	Divided attention, psychomotor and processing speed
Search birds with a certain color and form between an increasing number of distractors	300 s	Visuospatial function, processing speed
Stack blocks of numbers that differ by one on top of another to reduce the number of blocks	180 s	Planning
Remember the color and accessories of a penguin and at the same time the location of a fish	180 s	Working memory, processing speed
Finish a puzzle within a limited time	240 s	Visuospatial function, processing speed

#### Active control group

An active control condition is used to correct for the nonspecific cognitive activity that participants in the CT group go through. In the control condition, participants undergo cognitive engagement using three games (i.e. solitaire, trivia questions and hangman) with a total duration of 45 min that will sequentially be performed and are hypothesized not to train specific cognitive functions.

### Outcomes

#### Primary outcome

The primary outcome is the efficacy of CT on executive functions, measured by the percentage correct change score on a previously used computerized self-paced version of the Tower of London (ToL) task [29]. The ToL measures several aspects of executive functions, including planning, inhibition, and working memory [62]. This neuropsychological task consists of a model of three pins with different lengths, and three differently colored beads. In this task, the goal is to get from a starting position to a target position in as minimal steps as possible. There are five planning conditions that range in difficulty, with possible solutions ranging from one to five steps (i.e. task-load S1-S5). After nine exercise items with feedback, 100 pseudo-randomized test trials will be presented with a maximum response duration of 45 s per trial and no feedback on accuracy.

#### Secondary outcomes

The secondary outcome measures include (i) subjective cognitive complaints, (ii) cognitive function (other than the ToL) and (iii) structural and functional connectivity and brain network characteristics. All outcomes described below are changes after intervention relative to baseline.

i Subjective cognitive dysfunction change after the intervention will be measured by the Parkinson’s Disease Cognitive Functional Rating Scale (PD-CFRS, [63]) score and the Cognitive Failures Questionnaire (CFQ) score at the end of the intervention (T1), and at follow-up (T2, T3, and T4). We use the PD-CFRS questionnaire as a Parkinson-specific and sensitive measurement of subjective cognitive function. This questionnaire will be filled out by the participant and if possible by a caregiver. We will additionally use the CFQ as this measure has been used more frequently and it is more sensitive to small cognitive errors in daily living such as memory problems, absent-mindedness and slips of action [64];
ii Cognitive function change after the intervention will be measured by
  change on latent underlying cognitive factors in the neuropsychological assessment at T1 and at follow-up (T2, T3, and T4). Participants will undergo an extensive assessment battery of frequently-used and validated neuropsychological tests (see Table 2). See [65] for standard outcome measures of the neuropsychological tests. We will extract latent cognitive traits at baseline and measure training-induced changes on these factors at follow-up (see Analyses for a detailed description);
  reduction of the risk of developing PD-MCI or PD-D at follow-up at one-year and two-year follow-up. We will classify participants at the follow-up visits into level II PD-MCI [66] and probable PD-D [67] according to the most recent diagnostic criteria;
iii Training-induced neural alterations will be measured with magnetic resonance imaging (MRI). Morphometric brain characteristics will be measured with standard measures (i.e. subcortical volume, cortical thickness, fractional anisotropy). We will measure functional connectivity by extracting independent components of simultaneously fluctuating blood-oxygen level dependent signals that represent resting-state brain networks. Brain network characteristics will be measured by standard topological measures (i.e. modularity, global and local efficiency, betweenness centrality, see [68, 69]).

#### Exploratory outcomes and covariates

For exploratory purposes, the following outcomes will be collected.

- Training-induced cognitive changes on individual neuropsychological tasks (see Table 2) will be assessed to increase comparability with other CT studies, and to increase replicability of the results in future research;
- Improvement on the individual CT games will be measured in order to compare potential component-specific transfer effects. Performance on the CT components are collected automatically by the BrainGymmer online training module;
- Alterations on psychiatric symptoms of anxiety, depression, apathy, and impulse control disorders, using the Parkinson anxiety scale, Beck depression inventory, Apathy scale, and Questionnaire for Impulsive-Compulsive Disorders in Parkinson’s Disease – Rating Scale, respectively.

Additionally we will collect data on the following potential confounding factors:

- Data on physical activity at each visit will be measured by the New Zealand Physical Activity Questionnaire – Short Form, a structured interview on mild, moderate and vigorous physical activity, as physical activity is known to positively influence cognitive function and potentially provide a neuroprotective effect. [70, 71];
- We will rate motor symptom severity by the Unified Parkinson’s Disease – Rating Scale part III and assess disease stage by the modified Hoehn & Yahr stage [72];
- Medication usage data are collected and transformed into a ‘levodopa equivalent daily dosage’ [33]. Dopamine replacement therapy may influence cognitive functions [73, 74];
- Intervention compliance will automatically be monitored by the training module. We will calculate total compliance as the proportion of completed training games out of 24 total sessions: [N_completed_ / N_total_] × 100%, in which N_total_ is 13 games × 24 sessions in the CT condition, and 3 games × 24 sessions in the AC condition. We define non-compliance as a completion rate lower than 75%, in accordance with Petrelli and colleagues [75].

### Data-analyses

Data-analyses will be performed on the Modified-Intention-To-Treat population, which comprises the compliant participants that underwent at least 75% of the intervention and at least one post-training assessment. We will compare the baseline characteristics of this sample to the Intention-to-Treat population (all randomized subjects). Secondary Per Protocol-analyses will be performed comprising the population that underwent the complete study protocol. Analyses will be performed with IBM SPSS version 22 (Armonk, NY, USA) and in R [76]. We will employ a statistical threshold of α = .05.

The primary outcome will be analyzed using a multivariate mixed-model analysis using the accuracy on the five separate task-loadings (S1-S5) of the ToL at post-training visit (T1) as dependent measures, the training condition (CT vs. AC) as independent measure and baseline score of the outcome measures as covariates. We will construct a separate adjusted model with age, sex and years of education as additional covariates of no-interest. No imputation of missing values will be performed as this is not needed in linear mixed models.

The secondary outcome measures will also be analyzed with linear mixed-models with baseline score of the outcome measures as covariates. Subjective cognitive dysfunction will be modeled with the total score of the PD-CFRS (both self-report and caregiver) and the CFQ a) at post-training (T1) and b) at all follow-up assessments (T2, T3 and T4) as dependent variables. We will perform a factor analysis on all neuropsychological assessment outcomes (see Table 2) at baseline using a factor analysis with regularized maximum likelihood estimation to produce latent cognitive traits. We will compute baseline trait scores (i.e. factor scores), and compute trait scores at follow-up measurements based on the baseline factor analysis. The effect of CT on cognitive functions will be assessed with a multivariate mixed-model comparable to the above, using the trait scores as dependent variables. The effect of CT relative to AC on neuropsychiatric symptoms will be analyzed using similar multivariate mixed-models with as dependent variables the Beck Depression Inventory, the Parkinson Anxiety Scale, the Apathy Scale and the Questionnaire for Impulsive-Compulsive Disorders in Parkinson’s Disease – Rating Scale. Covariates will be added to the regression model based on a change-in-estimate method if there is a change of ≥10% of the regression coefficient for the intervention variable.

In order to analyze between-group differences in conversion to PD-MCI or PD-D, we will first classify patients at baseline, T3 and T4 as having normal cognition, PD-MCI or PD-D. We define conversion ‘down’ as conversion to a milder cognitive dysfunction classification, no conversion as classification in the same category at a later assessment visit and conversion ‘up’ as conversion to a worse cognitive function classification. We will assess the association between the intervention and conversion rate with a Fisher’s exact test. Odds ratios and confidence intervals of the conversion ‘down’ and no conversion groups versus the conversion ‘up’ group will be computed as a measure of effect size.

We will perform Fisher’s exact tests to verify if the demographic and clinical characteristics of the MRI subsample are similar to those of the full study sample. Functional MRI and diffusion tensor imaging data will be (pre) processed and analyzed with Statistical Parametric Mapping (SPM) software, FMRIB Software Library (FSL) and in-house Matlab (The MathWorks, Inc., Natick, MA, USA), scripts in combination with open-source toolboxes for (dynamic) network analysis [68, 69] to study the effects of cognitive training on the functional and structural brain network, respectively. We will also employ typical independent component analysis in combination with dual regression for resting-state functional connectivity and morphometric (e.g. cortical thickness) analysis on T1-weighted structural MRI to study within and between group-effects of our intervention. Moreover, to establish treatment response at the individual level, Multivariate Pattern classification (‘machine learning’) analyses will be performed to identify predictive markers (clinical, neuropsychological and neuroimaging) to be able to predict (in future patients) who is most likely to benefit from cognitive training.

#### Sample size

The sample size calculation is performed on the basis of a previous meta-analysis on the effects of CT on cognitive function [22]. This study showed an effect size of Hedges *g* = .23 (i.e. *f* = .12), based on the effect of CT on improving *global* cognitive function. The sample size needed to detect this effect is 112, based on a repeated-measures analysis of variance, corrected for a moderate correlation between pre- and post-treatment measures (i.e. *r* ≈ .6). This sample size estimation also provides a good indicator for the power of our multivariate mixed-model regression analysis with adjustment for baseline measures.

To ensure adequate power for the secondary study parameters, i.e. the development of PD-MCI and PD-D at one and 2 years follow-up, with an α = .05 and β = .8, and based on a small drop-out (~ 10%) given the home-based, easily-accessible training, we will include 140 participants.

## Discussion

The aim of the “COGnitive Training In Parkinson Study” (COGTIPS) is to assess the efficacy of an eight-week, online cognitive training program on alleviating cognitive dysfunction and subjective cognitive complaints, on delaying long-term cognitive deterioration and on increasing brain network connectivity and efficiency. COGTIPS is the first study in PD in a large group of PD patients –in accordance with recommendations from an earlier meta-analysis and review [22, 24]– that combines extensive clinical assessments with neuroimaging. We focus on PD patients in the ‘window of opportunity’, i.e., non-demented PD patients with mild subjective cognitive complaints that are expected to have the opportunity to employ significant neural plasticity in response to cognitive training. With the use of up to two-year follow-up assessments, this study can shed more light on the long-term effects of CT and its value in delaying conversion to PD-MCI and PD-D. The large subsample that will undergo MRI may show insight in the working mechanism of CT and baseline neuroimaging may additionally provide network organization characteristics that can predict individual training response.

The target population of COGTIPS consist of Dutch PD patients in the mild to moderate disease stage who experience significant subjective cognitive complaints but are not suspected of having PD-D. In this population that is often still active in work or social life, disease progression and cognitive decline provoke substantial worrying and are therefore an important subject of research [77]. The target population is large as about 50.000 Dutch individuals have PD, roughly 50% of whom have cognitive impairments [3], which does not include the even more prevalent subjective cognitive complaints that do not formally meet ‘impairment’ criteria [78]. However, the population is potentially heterogeneous given the large variety in age and degree of cognitive dysfunction. We may also expect ceiling scores on some of the neuropsychological assessment tasks in this non-demented PD population. We are, however, able to adhere to the level II criteria for PD-MCI and the criteria for probable PD-D using an extensive neuropsychological assessment battery [66, 67].

We will compare the CT adapted from the BrainGymmer environment to an active control condition based on ‘crystallized intelligence’ tasks. We thus correct for the cognitive engagement that participants are subjected to, to allow for any placebo effect mainly on subjective cognitive improvement and training effect on repeated cognitive assessment. Any CT-specific results will therefore be due to the training components. In the CT condition we will use an individually-based difficulty adaptation to adjust the training to the patients’ abilities. This ensures that participants are continuously stimulated at their own cognitive level and do not get frustrated or anxious by a training that is too difficult or bored by one that is too easy. Considering that we apply a home-based intervention and subjects can schedule their own training days, we expect a low attrition rate.

An important issue to overcome will be the medication use of participants, as the full study period will be more than 2 years. It is not realistic to expect stable medication over such a long period of time, although we will try to minimize medication changes as much possible in the first year by checking medication stability before subject participation and asking both the subject and neurologist to try and keep the medication regime stable. We will additionally correct for medication changes in our analyses and use a levodopa-equivalent daily dosage to aggregate the different types of PD medication.

There are substantial indications that cognitive training may provide an effective, non-pharmacological intervention to improve cognitive function in PD and delay cognitive decline, but evidence from large-scale RCTs is lacking. The aim of COGTIPS is to provide evidence for the efficacy of an easily-accessible, home-based online cognitive training, to validate the potential long-term effects and to shed more light on the underlying neural mechanism that mediate the beneficial effect of CT on cognitive function.

## Additional files

Additional file 1: Additional information. (PDF 237 kb)

Additional file 2: SPIRIT checklist of the COGnitive Training In Parkinson Study (PDF 183 kb)

## Data Availability

The datasets generated, used and analyzed during the COGTIPS trial and its preceding pilot trial are or will be available from the corresponding author upon reasonable request.

## Abbreviations

AC: Active control condition
COGTIPS: COGnitive Training In Parkinson Study
CT: Cognitive training
FSL: FMRIB Software Library
ICD: Impulse control disorder
MCI: Mild cognitive impairment
MRI: magnetic resonance imaging
PD: Parkinson’s disease
PD-CFRS: Parkinson’s Disease Cognitive Functional Rating Scale
PD-D: Parkinson’s disease dementia
RCT: Randomized controlled trial
SPM: Statistical Parametric Mapping
SPSS: Statistical Package for the Social Sciences
ToL: Tower of London
