# Supplementary material for: COGTIPS: a double-blind randomized active controlled trial protocol to study the effect of home-based, online cognitive training on cognition and brain networks in Parkinson’s disease
Source: BMC Neurol. 2019 Jul 31;19:179. doi: 10.1186/s12883-019-1403-6 (PMC6668056; doi:10.1186/s12883-019-1403-6)
Supplement: Supplementary file 1 — Additional information. (PDF 237 kb) [file 12883_2019_1403_MOESM1_ESM.pdf]

Additional file 1: Additional information

van Balkom et al. - COGTIPS: a double-blind randomized active controlled trial protocol to study the effect of home-based, online cognitive training on cognition and brain networks in Parkinson’s disease

Overview of cognitive assessments and questionnaires, including references

Cognitive screening

Montreal Cognitive Assessment [1]

Self-administered Gerocognitive Examination [2]

Neuropsychological tests

Boston naming test [3]

Category fluency [4]

Computerized adaptation of the Tower of London [5]

Controlled Oral Word Association Test (‘letter fluency’)[6]

Location Learning Test [7]

Pentagon copy from the Mini-Mental State Examination [8]

Rey Auditory Verbal Learning Test [9]

Rey Complex Figure Test [10]

Stroop Color Word Test [11]

Visual Form Discrimination Test [12]

Wechsler Adult Intelligence Scale-III digit span [13]

Questionnaires

Apathy scale [14]

Beck depression inventory

Alcohol and drug abuse screening (CAGE-AID, [15, 16])

Cognitive Failures Questionnaire [17]

Credibility/expectancy questionnaire [18]

New Zealand Physical Activity Questionnaire – Short Form [19]

Parkinson anxiety scale [20]

Parkinson’s Disease – Cognitive Functional Rating Scale [21]

Questionnaire for Impulsive-Compulsive Disorders in Parkinson's Disease – Rating Scale [22]

MRI parameters

All scans were acquired on a Discovery* MR750 3.0T MRI scanner (General Electric, Milwaukee) with a 32-channel head coil at the Amsterdam UMC, VU University (Amsterdam, the Netherlands).

Resting-state fMRI: 272 volumes (~10 minutes) of T2*-weighted echo-planar images (EPI's) with the following parameters: TR = 2200 ms, TE = 28 ms, flip angle = 80° , 42 axial slices (3.3 x 3.3 x 3.3 mm, matrix size 64 x 64). Sequential ascending acquisition according the hypophysis – fastigium (HYFA) line. High-order shimming (HOS) was performed to compensate for inhomogeneity in the magnetic field. Two reference scans in opposite phase-encode directions are acquired prior to the resting-state acquisition to correct for susceptibility induced distortions during post-processing: TR = 8000 ms, TE = 60 ms. The field-of-view, position, orientation and matrix dimensions are identical to the resting-state scan.

Diffusion-weighted MRI: Single Spin Echo multi-shell DWI with 73 diffusion weighted images (25 x b = 1000 s/mm^2, 24 x b = 2000 s/mm^2, 24 x b = 3000 s/mm^2) and seven non-diffusion weighted (b = 0 s/mm^2). TR = shortest (6000-7000 ms), TE = shortest (80-90 ms), 56 axial slices (2.5 x 2.5 x 2.5 mm, matrix size 128 x 128). Interleaved ascending acquisition according the hypophysis – fastigium (HYFA) line. High-order shimming (HOS) was performed to compensate for inhomogeneity in the magnetic field. Two reference scans in opposite phase-encode directions are acquired prior to the diffusion-weighted image to correct for susceptibility induced distortions during post-processing: TR = 8000 ms, TE = 60 ms. The field-of-view, position, orientation and matrix dimensions are identical to the diffusion-weighted image

Structural MRI: 3D sagittal MP-RAGE T1-weighted sequence according to ADNI-3 protocol with the following parameters: TI = 900 ms, TE = min full echo, flip angle = 8° 168 slices (1 x 1 x 1 mm, matrix size 256 x 256). 3D Cube sagittal Phase sensitive inversion recovery (PSIR) with the following parameters: TI = 650 ms , TR = 3000 ms, TE = minimum, 168 slices (1 x 1 x 1 mm, matrix size 256 x 256).

***Additional figure 1:** effect of cognitive training compared with an active control condition in a proof-of-concept.*

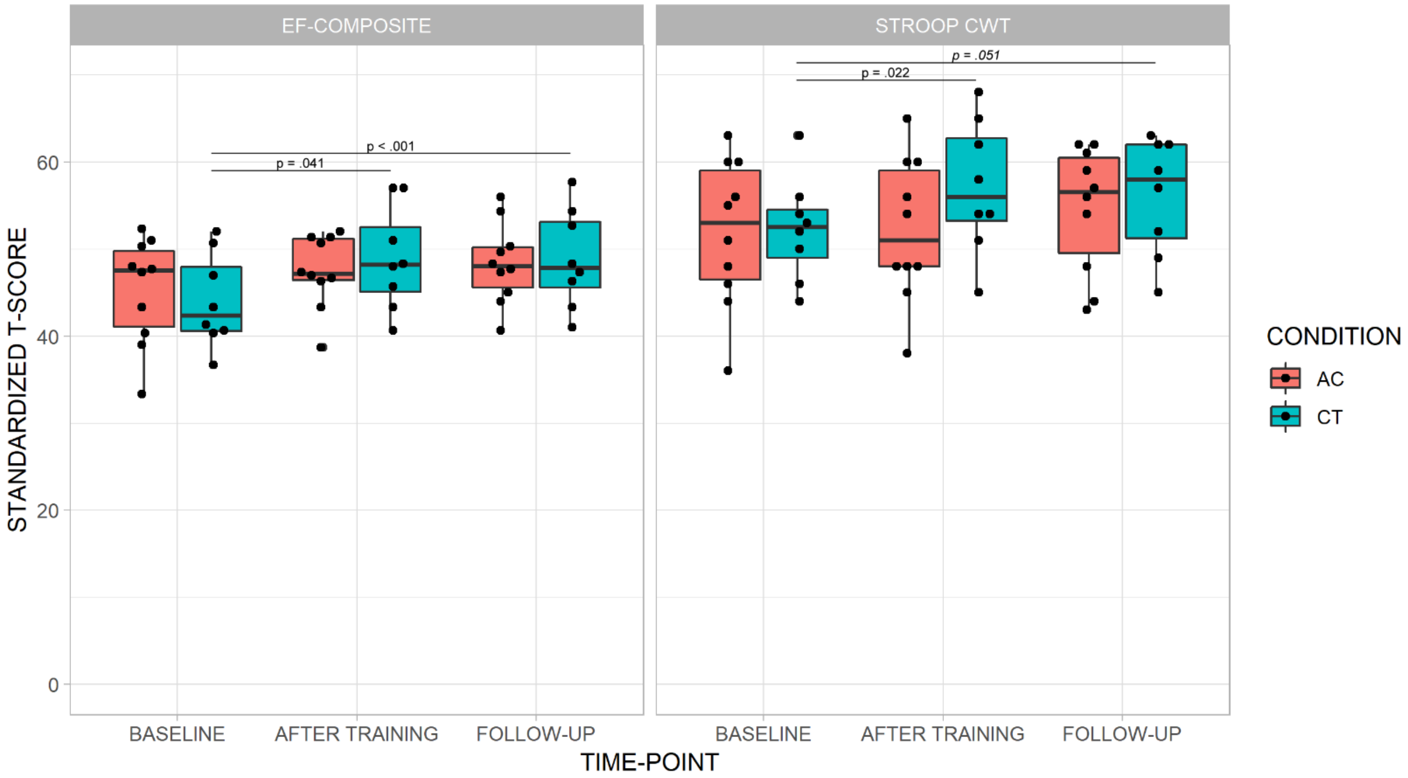

Additional figure 1 Change on median neuropsychological performance on left: an executive function composite score (consisting of standardized scores of the Stroop color word test card III corrected for card II, the Trail making test part B corrected for part A and the Controlled Oral Word Association Test) and right: the Stroop color word test card III corrected for card II. Significant differences are shown with the corresponding p value. Abbreviations: AC = active control condition; CT = cognitive training; EF = executive function; Stroop CWT = Stroop color word test.

References

1. Nasreddine ZS, Phillips NA, Bedirian V, Charbonneau S, Whitehead V, Collin I, Cummings JL, Chertkow H: **The Montreal Cognitive Assessment, MoCA: a brief screening tool for mild cognitive impairment**. *J Am Geriatr Soc* 2005, **53**(4):695-699.
2. Scharre DW, Chang SI, Murden RA, Lamb J, Beversdorf DQ, Kataki M, Nagaraja HN, Bornstein RA: **Self-administered Gerocognitive Examination (SAGE): a brief cognitive assessment Instrument for mild cognitive impairment (MCI) and early dementia**. *Alzheimer Dis Assoc Disord* 2010, **24**(1):64-71.
3. Kaplan E, Goodglass H, Weintraub S: **Boston naming test**: Pro-ed; 2001.
4. Luteijn F, Barelds D: **Herziening van de GIT. Handleiding bij de GIT-2**. In.: Harcourt Publishers, Amsterdam; 2005.
5. Shallice T: **Specific impairments of planning**. *Philos Trans R Soc Lond B Biol Sci* 1982, **298**(1089):199-209.
6. Schmand B, Groenink S, Van den Dungen M: **Letterfluency: psychometrische eigenschappen en Nederlandse normen**. *Tijdschr Gerontol Geriatr* 2008, **39**(2):64-74.
7. Kessels RP, Nys GM, Brands AM, van Zandvoort MJ: **[The Location Learning Test as a measure of spatial memory: applicability of a modified administration procedure and normative data]**. *Tijdschr Gerontol Geriatr* 2004, **35**(4):147-152.
8. Folstein MF, Folstein SE, McHugh PR: **"Mini-mental state". A practical method for grading the cognitive state of patients for the clinician**. *J Psychiatr Res* 1975, **12**(3):189-198.
9. Saan RJ, Deelman BG: **De 15-woordentest A en B (een voorlopige handleiding)**. Groningen: Afdeling Neuropsychologie, AZG; 1986.
10. Meyers JE, Meyers KR: **Rey Complex Figure Test and recognition trial professional manual**: Psychological Assessment Resources; 1995.
11. Hammes JGW: **De Stroop Kleur-Woord Test. Handleiding.** Amsterdam: Pearson Assessment and Information B.V.; 1971.
12. Benton AL, Sivan AB, deS Hamsher K, Varney NR: **Contributions to neuropsychological assessment: A clinical manual**: Oxford University Press, USA; 1994.
13. Wechsler D: **Wechsler adult intelligence scale - third edition. Nederlandstalige bewerking**. Amsterdam: Pearson Assessment and Information B.V.; 2000.
14. Starkstein SE, Mayberg HS, Preziosi TJ, Andrezejewski P, Leiguarda R, Robinson RG: **Reliability, validity, and clinical correlates of apathy in Parkinson's disease**. *J Neuropsychiatry Clin Neurosci* 1992, **4**(2):134-139.
15. Brown RL, Rounds LA: **Conjoint screening questionnaires for alcohol and other drug abuse: criterion validity in a primary care practice**. *Wis Med J* 1995, **94**(3):135-140.
16. Ewing JA: **Detecting alcoholism. The CAGE questionnaire**. *JAMA* 1984, **252**(14):1905-1907.
17. Broadbent DE, Cooper PF, FitzGerald P, Parkes KR: **The Cognitive Failures Questionnaire (CFQ) and its correlates**. *Br J Clin Psychol* 1982, **21 (Pt 1)**:1-16.
18. Devilly GJ, Borkovec TD: **Psychometric properties of the credibility/expectancy questionnaire**. *J Behav Ther Exp Psychiatry* 2000, **31**(2):73-86.
19. McLean G, Tobias M: **The New Zealand physical activity questionnaires: Report on the validation and use of the NZPAQ-LF and NZPAQ-SF self-report physical activity survey instruments.** . New Zealand: SPARC; 2004.
20. Leentjens AF, Dujardin K, Pontone GM, Starkstein SE, Weintraub D, Martinez-Martin P: **The Parkinson Anxiety Scale (PAS): development and validation of a new anxiety scale**. *Mov Disord* 2014, **29**(8):1035-1043.
21. Kulisevsky J, Fernandez de Bobadilla R, Pagonabarraga J, Martinez-Horta S, Campolongo A, Garcia-Sanchez C, Pascual-Sedano B, Ribosa-Nogue R, Villa-Bonomo C: **Measuring functional impact of cognitive impairment: validation of the Parkinson's disease cognitive functional rating scale**. *Parkinsonism Relat Disord* 2013, **19**(9):812-817.
22. Weintraub D, Mamikonyan E, Papay K, Shea Ja, Xie SX, Siderowf A: **Questionnaire for impulsive-compulsive disorders in Parkinson's Disease-Rating Scale**. *Mov Disord* 2012, **27**:242-247.
