# Supplementary material for: COGTIPS: a double-blind randomized active controlled trial protocol to study the effect of home-based, online cognitive training on cognition and brain networks in Parkinson’s disease
Source: BMC Neurol. 2019 Jul 31;19:179. doi: 10.1186/s12883-019-1403-6 (PMC6668056; doi:10.1186/s12883-019-1403-6)
Supplement: Supplementary file 2 — SPIRIT checklist of the COGnitive Training In Parkinson Study (PDF 183 kb) [file 12883_2019_1403_MOESM2_ESM.pdf]

Additional file 2: SPIRIT checklist of the COGnitive Training In Parkinson Study

van Balkom et al. - COGTIPS: a double-blind randomized active controlled trial protocol to study the effect of home-based, online cognitive training on cognition and brain networks in Parkinson's disease

SPIRIT 2013 Checklist

Section/item	ItemNo	Description	Comment or *in italics where information is reported*
**Administrative information**			
Title	1	Descriptive title identifying the study design, population, interventions, and, if applicable, trial acronym	*Administrative information*
Trial registration	2a	Trial identifier and registry name. If not yet registered, name of intended registry	*Abstract*
	2b	All items from the World Health Organization Trial Registration Data Set	*ClinicalTrials registry and full manuscript*
Protocol version	3	Date and version identifier	The most recent protocol version that is accepted by the Medical Ethical Committee of VUmc is dated 13-7-2018, version identifier 10
Funding	4	Sources and types of financial, material, and other support	*Declarations – Funding*
Roles and responsibilities	5a	Names, affiliations, and roles of protocol contributors	*Administrative information*
	5b	Name and contact information for the trial sponsor	*n.a. (investigator-initiated)*
	5c	Role of study sponsor and funders, if any, in study design; collection, management, analysis, and interpretation of data; writing of the report; and the decision to submit the report for publication, including whether they will have ultimate authority over any of these activities	*Declarations – Competing interests* and *Authors' contributions*

	5d	Composition, roles, and responsibilities of the coordinating centre, steering committee, endpoint adjudication committee, data management team, and other individuals or groups overseeing the trial, if applicable (see Item 21a for data monitoring committee)	*Procedure*
**Introduction**			
Background and rationale	6a	Description of research question and justification for undertaking the trial, including summary of relevant studies (published and unpublished) examining benefits and harms for each intervention	*Study objectives*
	6b	Explanation for choice of comparators	*Interventions*
Objectives	7	Specific objectives or hypotheses	*Study objectives*
Trial design	8	Description of trial design including type of trial (eg, parallel group, crossover, factorial, single group), allocation ratio, and framework (eg, superiority, equivalence, noninferiority, exploratory)	*Study design and setting*
**Methods: Participants, interventions, and outcomes**			
Study setting	9	Description of study settings (eg, community clinic, academic hospital) and list of countries where data will be collected. Reference to where list of study sites can be obtained	*Study design and setting*
Eligibility criteria	10	Inclusion and exclusion criteria for participants. If applicable, eligibility criteria for study centres and individuals who will perform the interventions (eg, surgeons, psychotherapists)	*Eligibility criteria*
Interventions	11a	Interventions for each group with sufficient detail to allow replication, including how and when they will be administered	*Interventions*

	11b	Criteria for discontinuing or modifying allocated interventions for a given trial participant (eg, drug dose change in response to harms, participant request, or improving/worsening disease)	Criteria for discontinuing the intervention will be a) the development of an impulse control disorder *during* the intervention, or b) initiated by the participant e.g. because of worsening disease or inability to adhere to the intervention schedule. Participants may withdraw from participation at any time, but will be motivated to continue participation if there is no medical reason not to. There will be no criteria for modifying the intervention for a given participant.
	11c	Strategies to improve adherence to intervention protocols, and any procedures for monitoring adherence (eg, drug tablet return, laboratory tests)	*Participant timeline – Eight-week intervention period*
	11d	Relevant concomitant care and interventions that are permitted or prohibited during the trial	Participants will be requested to retain a stable medication regime during the study period, specifically during the intervention. There are no further interventions prohibited during the trial.
Outcomes	12	Primary, secondary, and other outcomes, including the specific measurement variable (eg, systolic blood pressure), analysis metric (eg, change from baseline, final value, time to event), method of aggregation (eg, median, proportion), and time point for each outcome. Explanation of the clinical relevance of chosen efficacy and harm outcomes is strongly recommended	Outcomes

Participant timeline	13	Time schedule of enrolment, interventions (including any run-ins and washouts), assessments, and visits for participants. A schematic diagram is highly recommended (see Figure)	*Participant timeline, Figure 2* and *Table 2*
Sample size	14	Estimated number of participants needed to achieve study objectives and how it was determined, including clinical and statistical assumptions supporting any sample size calculations	*Data-analyses – sample size*
Recruitment	15	Strategies for achieving adequate participant enrolment to reach target sample size	*Discussion*

Methods: Assignment of interventions (for controlled trials)

Allocation:

Sequence generation	16a	Method of generating the allocation sequence (eg, computer-generated random numbers), and list of any factors for stratification. To reduce predictability of a random sequence, details of any planned restriction (eg, blocking) should be provided in a separate document that is unavailable to those who enrol participants or assign interventions	*Participant timeline – Condition allocation and instructions*
Allocation concealment mechanism	16b	Mechanism of implementing the allocation sequence (eg, central telephone; sequentially numbered, opaque, sealed envelopes), describing any steps to conceal the sequence until interventions are assigned	*Participant timeline – Blinding*
Implementation	16c	Who will generate the allocation sequence, who will enrol participants, and who will assign participants to interventions	*Participant timeline - Condition allocation and instructions*
Blinding (masking)	17a	Who will be blinded after assignment to interventions (eg, trial participants, care providers, outcome assessors, data analysts), and how	*Participant timeline - Condition allocation and instructions* and *Blinding*
	17b	If blinded, circumstances under which unblinding is permissible, and procedure for revealing a participant's allocated intervention during the trial	*Participant timeline – Blinding*

Methods: Data collection, management, and analysis

Data collection methods	18a	Plans for assessment and collection of outcome, baseline, and other trial data, including any related processes to promote data quality (eg, duplicate measurements, training of assessors) and a description of study instruments (eg, questionnaires, laboratory tests) along with their reliability and validity, if known. Reference to where data collection forms can be found, if not in the protocol	*Table 2* and *Additional file 1*
	18b	Plans to promote participant retention and complete follow-up, including list of any outcome data to be collected for participants who discontinue or deviate from intervention protocols	*Participant timeline – Post-intervention assessments* and *Drop-outs*
Data management	19	Plans for data entry, coding, security, and storage, including any related processes to promote data quality (eg, double data entry; range checks for data values). Reference to where details of data management procedures can be found, if not in the protocol	Data will be entered in an electronic CRF ('Castor electronic data capture') using coded data and double data entry and range checks if applicable.
Statistical methods	20a	Statistical methods for analysing primary and secondary outcomes. Reference to where other details of the statistical analysis plan can be found, if not in the protocol	*Data-analyses*
	20b	Methods for any additional analyses (eg, subgroup and adjusted analyses)	*Data-analyses*
	20c	Definition of analysis population relating to protocol non-adherence (eg, as randomised analysis), and any statistical methods to handle missing data (eg, multiple imputation)	*Data-analyses*

Methods: Monitoring

Data monitoring	21a	Composition of data monitoring committee (DMC); summary of its role and reporting structure; statement of whether it is independent from the sponsor and competing interests; and reference to where further details about its charter can be found, if not in the protocol. Alternatively, an explanation of why a DMC is not needed	This study will be monitored by the Clinical Research Bureau of the Amsterdam University Medical Centers, location VUmc – an independent monitoring party. Given the low estimated risk of this study, this study will be monitored with low intensity.
	21b	Description of any interim analyses and stopping guidelines, including who will have access to these interim results and make the final decision to terminate the trial	No interim analyses will be performed.
Harms	22	Plans for collecting, assessing, reporting, and managing solicited and spontaneously reported adverse events and other unintended effects of trial interventions or trial conduct	The risk of side effects or study-related adverse events in this study is negligible.<br>We will monitor the potential development of ICDs during the treatment by biweekly contact through e-mail of phone. Adverse events that are reported by patients or observed by the assessors will be monitored by the study team.
Auditing	23	Frequency and procedures for auditing trial conduct, if any, and whether the process will be independent from investigators and the sponsor	Two auditing visits will be conducted by the Clinical Research Bureau of the Amsterdam University Medical Centers, location VUmc (see 21a). This party is independent from the investigators.
**Ethics and dissemination**			
Research ethics approval	24	Plans for seeking research ethics committee/institutional review board (REC/IRB) approval	n.a. (study already approved by the VUmc Medical Ethical Committee, registration number 2016.543/NL58750.029.16)

Protocol amendments	25	Plans for communicating important protocol modifications (eg, changes to eligibility criteria, outcomes, analyses) to relevant parties (eg, investigators, REC/IRBs, trial participants, trial registries, journals, regulators)	Amendments will be sent to and evaluated by the VUmc Medical Ethical Committee
Consent or assent	26a	Who will obtain informed consent or assent from potential trial participants or authorised surrogates, and how (see Item 32)	Trained study members. A training log will be kept to ensure quality
	26b	Additional consent provisions for collection and use of participant data and biological specimens in ancillary studies, if applicable	Additional consent will be asked in the informed consent form
Confidentiality	27	How personal information about potential and enrolled participants will be collected, shared, and maintained in order to protect confidentiality before, during, and after the trial	All participant data will be coded. Personal information and the code key will be stored in a password-protected excel file. Written personal information will be stored separately from the data files.
Declaration of interests	28	Financial and other competing interests for principal investigators for the overall trial and each study site	*Declarations – Competing interests*
Access to data	29	Statement of who will have access to the final trial dataset, and disclosure of contractual agreements that limit such access for investigators	n.a. – this study is investigator-initiated. Data will be accessible for the investigator team.<br>Data and material is available upon reasonable request
Ancillary and post-trial care	30	Provisions, if any, for ancillary and post-trial care, and for compensation to those who suffer harm from trial participation	No ancillary and post-trial care provisions have been taken, but there is a liability insurance for all participants.

Dissemination policy	31a	Plans for investigators and sponsor to communicate trial results to participants, healthcare professionals, the public, and other relevant groups (eg, via publication, reporting in results databases, or other data sharing arrangements), including any publication restrictions	Trial results will be submitted to peer-reviewed journals. Results will be communicated to participants in a laymen summary.
	31b	Authorship eligibility guidelines and any intended use of professional writers	According to ICMJE criteria. No professional writers will be used.
	31c	Plans, if any, for granting public access to the full protocol, participant-level dataset, and statistical code	*Declarations - Availability of data and material*
**Appendices**			
Informed consent materials	32	Model consent form and other related documentation given to participants and authorised surrogates	Available upon request (in Dutch).
Biological specimens	33	Plans for collection, laboratory evaluation, and storage of biological specimens for genetic or molecular analysis in the current trial and for future use in ancillary studies, if applicable	n.a.
